# Cross-cultural adaptation and psychometric evaluation of the Chinese version of Childbirth Experience Questionnaire 2.0

**DOI:** 10.3389/fpsyt.2025.1488966

**Published:** 2025-06-20

**Authors:** Jiahui Wu, Yan Hong, Huici Guan, Mulan Huang, Jieqiong Liang, Zhimin Wen, Yulin Gao, Xiangang Feng

**Affiliations:** ^1^ Department of Psychiatry, The Fourth People’s Hospital of Shunde District (Shunde WuZhongpei Memorial Hospital), Foshan, China; ^2^ Jiangmen Maternity and Child Health Care Hospital, Jiangmen, China; ^3^ School of Nursing, Southern Medical University, Guangzhou, China; ^4^ School of Public Health, Southern Medical University, Guangzhou, China

**Keywords:** birth experience, childbirth experience questionnaire, puerperal women, psychometric evaluation, reliability, validity

## Abstract

**Background:**

Childbirth experience is a key determinant of maternal psychological well-being, and WHO emphasize promoting positive birth experiences. The Childbirth Experience Questionnaire (CEQ) is a widely used measure of women’s perceptions of labor and delivery. An improved version of this instrument, the CEQ 2.0, has not yet been adapted or psychometrically validated for use in mainland China. This study aimed to validate a Mainland version of CEQ 2.0 (CEQ 2.0-M) among Chinese postpartum women.

**Methods:**

A three-stage cross-sectional psychometric study was conducted among 700 postpartum women recruited from a tertiary hospital in mainland China (350 for EFA, 350 for CFA). Item analysis and dimensional refinement were applied to revise the original 25-item Chinese CEQ 2.0 before factor analyses. Structural validity was evaluated using parallel analysis, exploratory factor analysis (EFA), and confirmatory factor analysis (CFA). Reliability was assessed via Cronbach’s *α* and McDonald’s *ω*, and validity evidence included convergent, discriminant, concurrent, and known-group analyses.

**Results:**

In Stage 1, item analysis and theoretical review led to the refinement of the original Chinese CEQ 2.0, resulting in a four-factor version with improved conceptual clarity. In Stage 2, exploratory factor analysis supported this four-factor structure, explaining 53.2% of the total variance. Confirmatory factor analysis in Stage 3 indicated acceptable model fit (*χ* ^2^/*df* = 2.590; AGFI = 0.892; GFI = 0.927; CFI = 0.949; TLI = 0.934 and RMSEA = 0.068). Internal consistency was satisfactory, with Cronbach’s *α* = 0.65–0.91 and McDonald’s *ω* = 0.65–0.91 across subscales, and 0.84 and 0.80 respectively for the total scale. Evidence of convergent, discriminant, concurrent, and known-group validity further supported the scale’s psychometric robustness.

**Conclusions:**

The CEQ 2.0-M shows satisfactory psychometric properties and offers a valid, reliable instrument for assessing childbirth experiences among Chinese postpartum women. Its concise structure and established construct validity support its use in both clinical practice and research, particularly in developing countries seeking culturally appropriate tools for perinatal care evaluation.

## 1 Introduction

According to World Population Prospects data, approximately 130 million deliveries occurred in 2022 (1). Pregnancy and birth are important turning points in women’s lives. On the one hand, they bear significant physical discomfort and changes. Childbirth signifies a pivotal shift in social identity for primiparous women, while for multiparous women, it involves adapting to new family structures and re-evaluating their roles (2). Aligning with the new Global Strategy for Women’s, Children’s and Adolescents’ Health (2016–2030), attention is now paid not only to the survival rate of mothers and infants, but also to their abilities to maintain well-being (3). Therefore, WHO recommends greater attention to the importance of a “positive childbirth experience” (4).

However, different mothers have different childbirth experiences. Current research shows negative birth experiences are associated with increased risk of certain mental distress factors, such as postpartum depression (5) and postpartum PTSD (6). This leads to postpartum women feeling unsafety and losing control (7), and affects subsequent birth plans (8), mother-infant relationships (9), and other family relationships (10). In contrast, a positive birth experience can bring enjoyment and a sense of pride, and enhance abilities and expertise to raise infants (11).

Nevertheless, the birth experience is a complicated concept, consisting of many different dimensions and factors, including professional support (12), history of mental distress (13), fear of childbirth (14), stressful delivery (15), complications during pregnancy and birth (16), and so on. Previous research on childbirth experiences has predominantly focused on studying pathological psychological conditions (17), such as postpartum depression (5) and fear of childbirth (14). More recently, scholars have shifted their focus toward examining childbirth experiences from a positive perspective or considering the broader context (18). Antonovsky (19) introduced the Salutogenic Theory, which suggests that when focusing on health, people should not only consider the aspects related to disease, but also acknowledge the positive aspects and utilize all available resources to enhance health and prevent illness. Subsequently, Keyes (20) proposed that mental health is a holistic and continuous state, which means that when individuals strive to comprehend childbirth experiences, they need consider the positive, negative, and even neutral facets of these encounters.

Recognizing the intricate composition of childbirth experiences, a robust and culturally relevant measurement tool is imperative for a comprehensive assessment. The Childbirth Experience Questionnaire (CEQ) was first developed in 2010 to assess women’s multidimensional experiences of labor and birth (21). In 2020, a revised version (CEQ2.0) was released to address psychometric limitations identified in two of its original subscales (22). Both the original and revised versions have been widely translated and validated across countries and cultural settings (23–26). In 2016, Liao translated a 25-item manuscript version of the CEQ2.0 into simplified Chinese and conducted a validation study in mainland China, which reported good reliability and validity (27). Given that the 2016 version had already undergone expert review and preliminary cultural adaptation, and showed acceptable psychometric properties, it was adopted as the basis for the present study without repeating the translation process.

In 2022, Lok et al. validated the official CEQ2.0 in Hong Kong (CEQ2.0-R), but reported poor model fit in confirmatory factor analysis, which led to the exclusion of additional items to improve internal construct validity (28). Given that the Hong Kong sample represented a predominantly high-income population, the findings may not generalize to women from more diverse socioeconomic backgrounds. In mainland China, where regional economic disparities are considerable, it is important to assess childbirth experiences among medium- and low-income groups. This study aims to assess the psychometric properties of the existing simplified Chinese version of CEQ2.0 in mainland China (CEQ2.0-M), with potential implications for use in other developing countries.

## 2 Materials and methods

### 2.1 Participants

From February to October 2023, one cross-sectional study was conducted in Guangdong China. Postpartum women who had given birth within 3 to 5 days were recruited using convenience sampling by maternity ward nurses. To be eligible for the study, participants had to meet the following criteria: (1) be aged 18 or above; (2) be proficient in reading and writing Chinese. Exclusion criteria were: (1) significant illnesses such as malignant tumors; (2) unwillingness to participate or withdrawal from the study. These inclusion and exclusion criteria were intended to ensure that participants were physically and cognitively capable of completing the questionnaire, and to minimize potential recall bias due to medical complications.

The study was authorized by the Ethics Committee of the Jiangmen Maternity and Child Health Care Hospital which is a tertiary hospital in Guangdong, China (Protocol number: 2023022). All prospective participants received detailed information about the study and gave informed consent before joining the research voluntarily. Confidentiality of the participants was guaranteed throughout the study.

The CEQ2.0-M used in this study consists of 22 items. According to the COSMIN guideline recommending at least 10 participants per item, and assuming an 80% response rate, a minimum of 275 completed questionnaires was estimated to be required (29). Additionally, as one of the study objectives was to confirm strong construct validity through confirmatory factor analysis (CFA), a minimum sample size of 300 participants was required. Therefore, we needed at least 575 participants for this study. Nonetheless, as this study falls within a larger longitudinal research project which we secured a larger sample size for, we planned to use 350 participants (training sample) for item analysis and exploratory factor analysis (EFA), while allocating another 350 participants (validation sample) for CFA.

### 2.2 Measures

Birth experience was measured by the earlier Chinese version of CEQ2.0. The 22-item earlier Chinese version of CEQ2.0 evaluates childbirth experience, covering four dimensions: own capacity, perceived safety, professional support, and participation. Items are rated on a 4-point Likert-type scale (1 = totally disagree to 4 = totally agree). Using Visual Analogue Scales (VAS) to evaluate birth pain, the sense of control, and the safety of puerperal women. The VAS-scale scores were categorized into groups: 0-4 = 1, 5-6 = 2, 7-8 = 3, and 9-10 = 4. The total item score of higher means puerperal women has better childbirth experience. However, the scale includes items with reverse scoring. The simplified Chinese version used in this study was originally translated by Liao based on the 25-item manuscript of CEQ2.0, and had undergone expert review and preliminary cultural adaptation (27). Therefore, re-translation was not conducted. To align with the official 22-item version later published by Dencker et al., three items were removed (22). In addition, consultations with obstetric specialists were carried out to review the relevance of each item, resulting in a wording change from “midwife” to “healthcare professional.” This modification aligns more closely with the current childbirth context in the mainland and facilitates better understanding of the questionnaire content by maternal women.

### 2.3 Statistical methods

All data were entered using Epidata 3.1 and analyzed using SPSS 26.0, AMOS 24.0, and JASP 0.19.3. Data analysis was structured in three stages. In Stage 1, the reliability of the earlier 25-item Chinese version of CEQ2.0 was assessed using both Cronbach’s *α* and McDonald’s *ω*, followed by a confirmatory factor analysis (CFA) to test its original four-factor structure. In Stage 2, item analysis was conducted on the 22-item version of CEQ2.0-M, using item–total correlations and high–low group comparisons to assess item discrimination. An exploratory factor analysis (EFA) was conducted on the training sample to examine the latent factor structure and item performance, with the number of factors determined based on parallel analysis, theoretical framework, and factor loading patterns. In Stage 3, a confirmatory factor analysis (CFA) was performed on the validation sample to verify the revised structure and refine model fit using modification indices. Standardized factor loadings, average variance extracted (AVE), and composite reliability (CR), Cronbach’s *α*, and McDonald’s *ω* were computed to evaluate convergent validity and internal consistency, and known-group validity was further examined to assess the instrument’s ability to differentiate between predefined subgroups.

#### 2.3.1 Tests of reliability

Cronbach’s *α* coefficient was employed to assess the reliability and internal consistency of the scale. A Cronbach’s *α* value > 0.70 is generally considered as indicating good or acceptable reliability (30).

#### 2.3.2 Item analysis

Categorizing participants into high and low-scoring groups based on the top and bottom 27% of scores, respectively. First, a differential item functioning (DIF) analysis was performed on a training sample was employed using *t*-test. Items with a *t*-value < 3 were removed (31). Second, the remaining items were retained if their item-total correlation coefficient was ≥ 0.3 (30).

#### 2.3.3 Exploratory factor analysis

Prior to exploratory analysis, background equivalence between the training and validation samples was examined using independent-sample *t* tests and chi-square tests. EFA was conducted to determine structural validity. Before EFA, the Kaiser-Meyer-Olkin (KMO) value and Bartlett’s test of sphericity were checked for adequacy and correlation between items. The criteria for adequacy were KMO > 0.80 (32), and *p* < 0.001 for Bartlett’s test of sphericity (33). The number of factors to retain was determined using multiple criteria, including the scree plot, the Kaiser’s criterion (eigenvalues >1), and parallel analysis, in combination with the theoretical framework of the original CEQ2.0, which proposed a four-factor structure (26). Items with a factor loading < 0.4 were removed (31).

#### 2.3.4 Confirmatory factor analysis

CFA was conducted to test the new model derived from the prior EFA. The model fit of the CFA model was assessed by the following indices and their corresponding cut-off criteria: *χ* ^2^/*df* < 3; adjusted goodness of fit index (AGFI) > 0.85; goodness of fit index (GFI) > 0.85; comparative fit index (CFI) > 0.90; root mean square error of approximation (RMSEA) < 0.08 and Tucker-Lewis index (TLI) ≥ 0.90 (34–39). The convergent validity and reliability of the model were evaluated by the average variance extracted (AVE) and composite reliability (CR), with the criteria of AVE > 0.5 and CR > 0.8 (31).

#### 2.3.5 Concurrent validity

Concurrent validity was assessed in this study by collecting data on participants’ satisfaction with childbirth alongside the CEQ2.0-M scale. A question was included that directly inquired about their satisfaction with childbirth experience using a continuous 0 to 10 scale with higher scores indicating greater satisfaction. Given the ordinal distribution and skewness of CEQ2.0-M scores, Spearman’s rank-order correlation was used to examine associations between the total and subscale scores of CEQ2.0-M and the satisfaction rating. Concurrent validity was determined by correlating the satisfaction scores with the overall and subscale scores of the CEQ2.0-M.

#### 2.3.6 Known-group validity

Known-group validity was examined to determine whether the CEQ2.0-M could distinguish between subgroups based on demographic and obstetric characteristics. Non-parametric tests were used due to non-normal distribution of the CEQ2.0-M scores. Mann–Whitney U test was used for comparisons between two groups; Kruskal–Wallis H test was applied for multi-category variables. Statistical significance was set at α = 0.05(two-tails).

## 3 Result

### 3.1 Participant characteristics

**Table 1** presents the demographic and obstetric characteristics of the 700 puerperal women, divided into a training sample (*n* = 350) and a validation sample (*n* = 350). Participants’ average age was 30.2(4.6). The unit of Monthly Family Income is in Chinese Yuan (CNY), with an exchange rate of approximately 1 US Dollar ≈ 7.2 Chinese Yuan as of November 2023. No significant differences were found between the training and validation samples in terms of maternal age, monthly family income, education level, parity, or mode of birth, indicating good comparability between the two groups.

**Table 1:** Sample characteristics (*N* = 700).

Characteristics	Total	Training sample	Validation sample	*t/χ^2^*	*p*
	*N* = 700	*n* = 350	*n* = 350		
Maternal age, years, mean (SD)	30.2 (4.6)	30.2 (4.7)	30.2 (4.6)	0.242	0.809
Monthly family income (CNY),				2.827	0.587
< 3,000	31 (4.6)	15 (4.4)	18 (5.2)		
3,000 – 4,999	101 (15.0)	58 (17.1)	44 (12.8)		
5,000 – 9,999	286 (42.4)	138 (40.6)	151 (44.0)		
10,000 – 20,000	201 (29.8)	100 (29.4)	102 (29.7)		
> 20,000	56 (8.3)	29 (8.5)	28 (8.2)		
Education, *n* (%)				0.002	0.969
Secondary school or below	286 (41.8)	144 (41.6)	147 (42.5)		
College or above	398 (58.2)	202 (58.4)	199 (57.5)		
Mode of birth, *n* (%)				2.904	0.407
Spontaneous vaginal birth	427 (61.8)	217 (62)	215 (61.6)		
Assisted vaginal birth	19 (2.7)	12 (3.4)	7 (2.0)		
Planned caesarean	128 (18.5)	58 (16.6)	72 (20.6)		
Emergency cesarean	117 (16.9)	63 (18.0)	55 (15.8)		
Parity, *n* (%)				0.648	0.421
Primiparous	432 (62.4)	223 (63.9)	214 (61.5)		
Multiparous	260 (37.6)	126 (36.1)	134 (38.5)		

*Missing values for each variable do not exceed 3.6%.

*Missing values indicate incomplete data and were excluded from statistical analysis for this variable.

### 3.2 Reliability and validity assessment of the earlier Chinese version of CEQ2.0

The Cronbach’s *α* and McDonald’s *ω* coefficients for the subdimensions were as follows: Own Capacity, 0.69 (*α*) and 0.71 (*ω*); Professional Support, 0.41 (*α*) and 0.69 (*ω*); Perceived Safety, 0.71 (*α*) and 0.74 (*ω*); and Participation, 0.24 (*α*) and 0.33 (*ω*). The overall reliability coefficients for the scale were 0.80 for both Cronbach’s *α* and McDonald’s *ω*.

The earlier Chinese version of CEQ2.0 fit the data into the model using confirmatory factor analysis (CFA). However, the model did not fit well with the data (*χ* ^2^/*df* =4.170; GFI=0.78; AGFI=0.74; CFI=0.73; IFI=0.73; RMSEA=0.09, **Table 2**). Therefore, a psychometric evaluation of the earlier Chinese version of CEQ2.0 is necessary.

**Table 2:** Goodness of fit indicators of the test for both the earlier Chinese version of CEQ2.0 factor model and CEQ2.0-M factor model.

Model	*N*	*χ* ^2^/*df*	GFI	AGFI	CFI	IFI	TLI	RMSEA
Acceptable value	/	<3.000	>0.850	>0.850	>0.900	>0.900	>0.900	<0.080
the earlier Chinese version of CEQ2.0	350	4.170	0.781	0.736	0.726	0.729	0.695	0.095
CEQ2.0-M: 3-factor model	350	4.455	0.874	0.826	0.870	0.871	0.843	0.100
CEQ2.0-M: 4-factor model	350	2.590	0.927	0.892	0.949	0.949	0.934	0.068

*χ*²/*df*, chi-square divided by degrees of freedom; GFI, goodness-of-fit index; AGFI, adjusted goodness-of-fit index; CFI, comparative fit index; IFI, incremental fit index; TLI, Tucker–Lewis index; RMSEA, root mean square error of approximation.

CEQ2.0-M models were developed based on either parallel analysis (3-factor) or theoretical structure (4-factor).

### 3.3 Refinement and psychometric evaluation of the CEQ2.0-M

#### 3.3.1 Item reduction

The total 22-item scores range from 28 to 80. After conducting independent sample *t*-tests on the high and low-scoring groups, items with *t*-values less than 3 were excluded, including item 5 and 9. While it was found that item 15 and 16 scored lower in the high-scoring group than in the low-scoring group, they were also removed. After calculating the item-total correlations, it was found that item 23 had correlations less than 0.3. Therefore, 17 items were retained for further psychometric evaluation.

#### 3.3.2 Structural validity

The Kaiser–Meyer–Olkin (KMO) measure verified the sampling adequacy for the analysis, with an overall MSA of 0.862. Bartlett’s test of sphericity was significant (*χ*² = 2692.06, *df* = 136, *p* < 0.001), indicating sufficient correlations among items for conducting factor analysis. Parallel analysis indicated a three-factor solution. Parallel analysis suggested a three-factor solution, as only the first three eigenvalues exceeded the simulated values. However, the fourth eigenvalue (1.14) was only slightly below the simulated threshold (1.20), suggesting a borderline factor. Considering the theoretical four-factor structure of the original CEQ2.0 and the conceptual clarity of the extracted factors, a four-factor solution was retained. To further support this decision, confirmatory factor analyses were subsequently conducted to compare the model fit of both three- and four-factor structures.

Exploratory factor analysis was conducted using principal axis factoring with oblimin rotation to allow for correlations among factors. After oblimin rotation, the four-factor solution accounted for 53.2% of the total variance. Items CEQ3, CEQ12, and CEQ20 were removed due to low factor loadings (< 0.40), resulting in a 14-item solution. After removing these items, the final four-factor solution accounted for 58.0% of the total variance, indicating a modest improvement in explained variance. Finally, 14-items were retained, including 5 items for “Professional Support”, 5 items for “Own Capacity”, 2 items for “Negative Emotion”, and 2 items for “Perceived Safety” (**Table 3**).

**Table 3:** Rotated factor matrix of the CEQ2.0-M.

Factor	Item	Factor loading			
		F1	F2	F3	F4
Professional Support					
19	My impression of the team’s medical skills made me feel secure.	0.866			
18	The midwife helped me to find my inner strength.	0.865			
17	The midwife conveyed an atmosphere of calm.	0.861			
14	I received the information I needed during labor and birth.	0.755			
13	Both my partner and I were treated with warmth and respect.	0.700			
Own Capacity					
2	I felt strong during labor and birth.		0.768		
7	I felt that I handled the situation well.		0.698		
4	I felt capable during labor and birth.		0.624		
6	I felt happy during labor and birth.		0.424		
1	Labor and birth went as I had expected.		0.419		
Negative Emotion					
22	Some of my memories from childbirth make me feel depressed. ^R^			0.895	
21	I have many negative memories from childbirth. ^R^			0.781	
Perceived Safety					
24	As a whole, how much control did you feel you had during childbirth? ^a^				0.712
25	As a whole, how secure did you feel during childbirth? ^a^				0.590
Eigenvalue		5.911	2.102	1.623	1.143
Percent of variance of Rotation Sums of Squared Loadings		21.50%	13.40%	9.70%	8.50%
Total percent of variance explained		53.20%			

Extraction method: Principal Axis Factoring. Rotation method: Oblimin rotation. The number of factors was determined based on parallel analysis.

Items were removed due to low factor loadings: “I took part in decisions regarding my care and treatment as much as I wanted,” “I have many positive memories from childbirth,” and “I felt scared during labor and birth.”

a Visual analogue scale (VAS); ^R^ Reversed item: Reversely coded before inclusion in the analysis.

Confirmatory factor analyses were conducted to examine the model fit of both the three-factor and four-factor structures derived from the exploratory analyses. The three-factor model, as suggested by parallel analysis, showed suboptimal fit indices (*χ* ^2^/*df* = 4.455; AGFI = 0.826; GFI = 0.874; CFI = 0.870; TLI = 0.843 and RMSEA = 0.100), indicating a poor fit to the data. In contrast, the four-factor model based on the theoretical structure of the original CEQ2.0 and the revised item configuration showed satisfactory fit (*χ* ^2^/*df* = 2.590; AGFI = 0.892; GFI = 0.927; CFI = 0.949; TLI = 0.934 and RMSEA = 0.068). These results confirmed the structural validity of the four-factor model of the CEQ2.0-M, which was subsequently adopted as the final model (**Table 2**).

#### 3.3.3 Convergent validity and discriminant validity

Based on the study, all the subscales of the CEQ2.0-M had a significant and high positive correlation with the total score. This indicates adequate convergent validity. Specifically, higher childbirth experience score meant the puerperal women felt higher professional support (*r* =0.68; *p* < 0.001), felt more confident in their own capacity (*r* =0.83; *p* < 0.001), felt less negative emotion (*r* =0.64; *p* < 0.001), had higher perceived safety (*r* =0.68; *p* < 0.001). The scale suggests a reasonable level of discriminant validity, considering that the correlations between each dimension do not exceed the square root of their respective AVE values.

#### 3.3.4 Concurrent validity

Results showed that higher satisfaction with childbirth was significantly correlated with higher CEQ2.0-M score (*r* =0.54; *p* < 0.001), felling more confident in their Own Capacity (*r*=0.50; *p* < 0.001), having higher Perceived Safety (*r* =0.41; *p* < 0.001), felling higher Professional Support (*r* =0.32; *p* < 0.001), felling less Negative Emotion (*r* =0.29; *p* < 0.001). These findings suggest adequate concurrent validity of the CEQ2.0-M scale.

#### 3.3.5 Known-group validity

To assess known-group validity, CEQ2.0-M scores were compared across subgroups defined by obstetric characteristics using Mann–Whitney U and Kruskal–Wallis H tests. Significant differences in total scores of CEQ2.0-M were observed across subgroups defined by maternal age (*p* < 0.001), education level (*p*=0.566), mode of birth (*p*=0.015), parity (*p* < 0.001), perineal laceration (*p* = 0.074), and companionship during labor (*p* = 0.852). Additionally, subgroup differences were found at the subscale level. Education level was associated with differences in Own Capacity (*p* = 0.014), and women with perineal laceration scored significantly higher in Negative Emotion (*p* = 0.042) and professional support (*p* < 0.001). No significant differences in CEQ2.0-M scores were found across subgroups defined by preterm birth status. Although companionship during labor was analyzed, no significant differences were found at the subscale level, and the variable was retained in the table for completeness. Detailed results for total and subscale comparisons are presented in **Table 4**.

**Table 4:** Known-group validity of the CEQ2.0-M: Differences in domain scores and total score across demographic and obstetric variables. (*N*=700).

Group	*n*	CEQ-E 2.0 Total Score	Domain 1 Professional support	Domain 2 Own Capacity	Domain 3 Negative Emotion	Domain 4 Perceived Safety
		*M*(SD)	*M*(SD)	*M*(SD)	*M*(SD)	*M*(SD)
Maternal age						
<35 years	578	43.62(6.32)	17.55(2.53)	14.89(2.93)	5.63(1.77)	5.54(1.65)
≥35 years	113	46.46(6.69)	17.99(2.43)	16.32(2.93)	6.13(1.83)	6.03(1.48)
*p*-value		< 0.001	0.087	< 0.001	0.003	0.003
Education						
Secondary school or below	286	44.25(6.60)	17.49(2.55)	15.43(3.13)	5.59(1.85)	5.74(1.67)
College or above	398	43.94(6.37)	17.72(2.50)	14.91(2.86)	5.78(1.74)	5.53(1.60)
*p*-value		0.566	0.170	0.014	0.187	0.154
Mode of birth						
Spontaneous vaginal birth	427	44.64(6.27)	17.72(2.60)	15.22(2.86)	5.87(1.71)	5.84(1.57)
Assisted vaginal birth	19	40.55(7.24)	16.95(2.25)	13.29(3.97)	5.05(1.75)	5.26(1.59)
Planned caesarean	128	43.74(6.29)	17.30(2.44)	15.59(2.69)	5.51(1.86)	5.34(1.62)
Emergency cesarean	117	42.99(6.92)	17.75(2.32)	14.58(3.37)	5.46(1.95)	5.20(1.75)
*p*-value		0.015	0.120	0.020	0.026	< 0.001
Parity						
Primiparous	432	42.58(6.49)	17.36(2.56)	14.26(2.90)	5.70(1.65)	5.27(1.62)
Multiparous	260	44.99(6.28)	17.79(2.48)	15.65(2.90)	5.72(1.86)	5.84(1.60)
*p*-value		< 0.001	0.017	< 0.001	0.626	< 0.001
Perineal laceration						
No	354	43.63(6.68)	17.55(2.37)	15.10(3.12)	5.58(1.84)	5.39(1.69)
Yes	338	44.57(6.19)	17.71(2.66)	15.15(2.82)	5.85(1.72)	5.87(1.54)
*p*-value		0.074	0.198	0.909	0.042	< 0.001
Companionship during labor						
No	131	44.40(6.50)	17.41(2.53)	15.32(2.90)	5.95(1.71)	5.73(1.66)
Yes	556	44.03(6.47)	17.70(2.52)	15.08(3.00)	5.66(1.80)	5.59(1.63)
*p*-value		0.852	0.180	0.469	0.117	0.431

M, mean; SD, standard deviation. *p*-values were calculated using Mann–Whitney U or Kruskal–Wallis H tests. Missing data per variable did not exceed 1.5% of the total sample.

#### 3.3.6 Reliability

The overall scale showed acceptable reliability with *α* = 0.84 and *ω* = 0.80. For the subscales, the Cronbach’s *α*/McDonald’s *ω* values were as follows: Professional Support: 0.91/0.91; Own Capacity: 0.78/0.78; Negative Emotion: 0.84/0.84; Perceived Safety: 0.65/0.65. While most subscales showed satisfactory internal consistency, the Perceived Safety subscale showed relatively lower reliability, which may be due to the limited number of items (*n* = 2). As the data were collected within five days after delivery to reduce recall bias, test–retest reliability was not conducted.

## 4 Discussion

Childbirth experience has a lasting impact on maternal physical and mental health, and robust measurement is essential for designing effective clinical interventions. Such studies therefore require instruments with sound psychometric properties to capture childbirth experience accurately. CEQ2.0, as a tool for measuring childbirth experience, has been translated and used in multiple countries—showing generally sound psychometric performance (26, 40, 41). However, evidence from mainland China and other developing Asian settings remains limited. Building on the earlier 25-item Chinese adaptation of the CEQ 2.0, the present study developed and validated a 14-item version (CEQ 2.0-M) through item analysis, confirming a robust four-factor structure with satisfactory reliability and construct validity. These findings support the use of the CEQ 2.0-M for both clinical screening and future research in Asian settings and other developing, low- and middle-income contexts.

Validity evidence for the CEQ 2.0-M encompassed structural validity (including parallel analysis and CFA), convergent validity, discriminant validity, concurrent validity, and known-group validity. Reliability was examined via item–total correlations, Cronbach’s *α* and McDonald’s *ω*. The results suggest that after the psychometric evaluation, the CEQ2.0-M shows good reliability and validity across all aspects.

As shown by the suboptimal reliability indices and inadequate CFA fit of the original 25-item Chinese CEQ 2.0, its internal consistency proved insufficient—a limitation also observed in the Hong Kong adaptation (28), underscoring the need for structural refinement. We therefore conducted systematic item analysis followed by EFA and CFA to derive a refined structure. The resulting 14-item version preserved the original four theoretical domains, with items selectively removed based on psychometric performance. Own Capacity and Professional Support remained largely intact, Perceived Safety was modestly refined, while the underperforming Participation items were omitted and replaced by a Negative Emotion domain. Although this trajectory parallels the Hong Kong adaptation, our domain labels and item composition differ in three of the four factors, underscoring contextual nuances that future cross-cultural work should address (28).

### 4.1 Discussion of dimensions

While parallel analysis initially supported three factors, the fourth eigenvalue was marginally below the threshold and the fourth domain remained conceptually meaningful (22). Combined with superior CFA fit for the four-factor model, this justified retaining all four domains in the CEQ 2.0-M.

Factor 1 was labeled “Professional Support” and included items reflecting interactions between puerperal women, their families and healthcare professional. It also covers some significant elements like compassion, understanding, dignity, and respect—consistent with the original conceptualization (22). Consistent with previous studies, the quality of professional support is a key determinant of childbirth experience (42). Effective support extends beyond verbal communication alone (12). It implies that healthcare professional should be conscious of the fact that their attitudes toward childbirth can also influence women’s perceptions of their childbirth experience (43). This helps healthcare professional contemplate their role orientation (44) and adjust their approach during labor and delivery. Removing items about accompanies and encouragement from healthcare professional may be because these items couldn’t precisely measure the actual support during childbirth. Research shows that one-third of women feel the support from midwives doesn’t meet their expectations, possibly due to high expectations and limited healthcare resources. These findings underscore the need to clarify and strengthen midwifery support, particularly in resource-limited settings (45). Known-group analyses indicated that Professional Support scores differed significantly only by parity in our sample, whereas the Icelandic validation found differences by age, income, parity, and birth mode. Both studies reported no education-level effects, suggesting that cultural and healthcare system factors may modulate subgroup influences on perceived professional support (25).

Factor 2, labeled “Own Capacity,” includes items reflecting women’s perceived self-efficacy—such as feelings of empowerment, positive anticipation, and emotional uplift (22). These appear related to puerperal women’s self-efficacy. This accords with earlier studies indicating that enhanced self-efficacy of puerperal women can improve birth experiences and perinatal outcomes (46). Meeting women’s expectations raises their birth satisfaction, so helping set flexible anticipation, assists this (47). Healthcare professionals should also address emotional needs during delivery and labor to indirectly improve birth satisfaction (48). Moreover, expressing trust in women’s capabilities further empowers them throughout childbirth (49). Through item analysis, the statement “I was tired during labor and birth” showed poor discrimination and was thus removed after expert review, as fatigue is a ubiquitous labor experience. Known-group analyses indicated that Own Capacity scores differed significantly by maternal age, education level, mode of birth, and parity in our sample, whereas perineal laceration and birth companionship showed no effect. In contrast, the Swedish validation observed significant differences only for parity and birth mode, while the Icelandic study reported subgroup effects for age, education, parity, and birth mode. These variations likely reflect differences in cultural expectations and maternity care practices across settings (25, 41).

Factor 3 “Negative Emotion” covers fear of birth, depression, and negative memories. This is a new factor identified through exploratory analysis, though the original study classified these under “Perceived Safety” (22). This aligns with findings that higher negative emotion scores are associated with poorer childbirth experiences (50). Although these negative emotions do not necessarily indicate clinical disorders, they still merit attention alongside established concerns of postpartum depression and PTSD (17). Known-group analyses revealed that Negative Emotion scores differed significantly by maternal age, mode of birth, and the presence of perineal laceration.

Factor 4 “Perceived Safety” includes items relevant to puerperal women’s sense of security and control during delivery and labor (22). Previous research shows heightened feelings of security and trust promotes a sense of internal control associated with positive birth experiences (51). It’s worth noting that both the CEQ2.0-R(Hong Kong version) and the CEQ2.0-M excluded the dimension of “participation” which was newly developed in the CEQ2.0 (22, 28). This suggests Chinese women typically make decisions about the method of childbirth based on the advice and guidance provided by healthcare professionals (52). Known-group analyses showed significantly lower Perceived Safety scores among younger mothers, women undergoing operative or emergency births, preterm deliveries, and those with perineal laceration, underscoring the domain’s discriminant validity. In contrast with the Hong Kong revision—whose *Perceived Safety* items center on negative post-birth memories (“I have many negative memories from childbirth”; “Some of my memories from childbirth make me feel depressed”)—our CEQ 2.0-M operationalizes this domain through immediate feelings of control and security during labor (“As a whole, how much control did you feel you had during childbirth?”; “As a whole, how secure did you feel during childbirth?”). This shift from retrospective affect to real-time appraisal may account for the different subgroup patterns observed between the two studies.

### 4.2 Strengths and limitations

A key strength of the CEQ 2.0-M is its concise 14-item format, enhancing feasibility for postpartum women who may experience fatigue with longer instruments. Secondly, following psychological measurement assessments among individuals with low to moderate incomes, the questionnaire could be considered for broader application in developing countries. Despite these strengths, several limitations should be noted. The sample was recruited from third-tier cities in China, and specific environmental factors may constrain generalizability of the findings to other regions or contexts. Additionally, “Negative emotion” and “Perceived safety” subscales have fewer items, which may marginally impact reliability. Test–retest reliability was not assessed to minimize recall bias, representing another limitation to be addressed in future studies.

## 5 Conclusion

This study showed that the 14-item, 4-domain Chinese version of the Childbirth Experience Questionnaire (CEQ2.0-M) has adequate psychometric properties for effectively assessing childbirth experiences of postpartum women. The CEQ2.0-M domains are, “Professional Support” (5 items), “Own Capacity” (5 items), “Negative Emotion” (2 items), and “Perceived Safety” (2 items). This study suggested that the CEQ2.0-M has good reliability and validity.

In clinical practice, the CEQ2.0-M provides valuable insights into postpartum women’s childbirth experiences and enables timely interventions based on identified needs. Furthermore, healthcare professionals can use patients’ CEQ2.0-M scores to evaluate if adequate professional and emotional support was provided throughout the labor and delivery process, aiding in assessments of care quality and compassion. Future research with diverse populations and healthcare settings is recommended to further establish generalizability of the scale.

## Data Availability

The datasets for this article are not publicly available due to participant privacy concerns and the protection mandated by the Ethics Committee of the Jiangmen Maternity and Child Health Care Hospital. Requests to access the datasets should be directed to jmbjykjk@163.com.
